# Determinants of depressive and alcohol use disorders among female sex workers in Ethiopia: evidence from a national bio-behavioral survey, 2020

**DOI:** 10.1186/s12888-024-05799-9

**Published:** 2024-05-07

**Authors:** Jemal Ayalew Yimam, Sileshi Luslseged, Jaleta Bulti Tura, Birra Bejiga Bedassa, Feyiso Bati Wariso, Mohammed Ahmed Rameto, Saro Abdella

**Affiliations:** 1 Department of statistics, College of Natural Science, Wollo University, Kombolcha, Ethiopia; 2 College of Public Health, Addis Ababa University, Addis Ababa, Ethiopia; 3 TB/HIV Directorate, Ethiopian Public Health Institute, Addis Ababa, Ethiopia

**Keywords:** Severe depression, FSWs, Alcohol dependent, Bivariate ordinal data analysis, RDS, Ethiopia

## Abstract

**Background:**

Female sex workers (FSWs) face an elevated risk of developing mental health disorders and alcohol use disorders (AUD), which in turn increase their vulnerability to HIV and other sexually transmitted infections (STIs) and other negative outcomes. To effectively address both of these health issues, it is crucial to understand the shared key determinants underlying these illnesses, which is a substantial knowledge gap in Ethiopia and elsewhere in the world. Therefore, this study aimed to identify the common key determinants of depression and AUD among FSWs in Ethiopia using a bivariate multivariable ordinal logistic model.

**Methods:**

We analyzed cross-sectional biobehavioral data collected in 2020 from 16 cities and major towns in Ethiopia using the respondent-driven sampling (RDS) technique, which involved a total of 6,085 FSWs. FSWs who had lived at the study sites for at least a month before the study period were deemed eligible for recruitment. Major depressive disorder (DD) and AUD were screened using the Patient Health Questionnaire (PHQ9) and alcohol use disorder identification test (AUDIT), respectively. We used descriptive statistics to summarize study population characteristics and bivariate multivariable ordinal logistic regression (BMOLR) to identify common determinants of DD and AUD combined and their nonnormal correlation.

**Results:**

Among 6085 FSWs screened for DD and AUD, 13.5% and 4.0% have met the criteria for moderate and severe depressive disorder, respectively, and 20.3% and 34.7% have met the AUDIT criteria for harmful or hazardous behavior and alcohol dependence, respectively. FSW with experience of inconsistent condom use, condom failure, violence, mobility, use of any drugs, non-paying partners, abortion, and selling sex for more than five years were associated with an increase in the severity of both disorders. A high average income from selling sex and the number of paying partners reduced the severity of depression and increased the level of alcohol dependence. Being HIV positive and ever having anal sex were associated only with an increase in depression.

**Conclusion:**

Major DD and AUD are prevalent among FSWs in Ethiopia. The findings revealed that common key determinants, which exacerbated the severity of both disorders, were also risk factors for HIV and other STIs. Consequently, integrated STI strategies are essential in the screening, referral, and treatment of depression and AUD. Intervention packages should encompass determinants of depression and AUD, including condom utilization, drug use, mobility between towns, abortion, violence, and counseling services. Additionally, strategies to ensure economic security should be incorporated.

## Background

Worldwide, depressive disorder (DD) and alcohol use disorder (AUD) are among the most prevalent psychiatric problems [1, 2], contributing to 14% of the global burden of disease [3, 4]. These disorders co-occur frequently [4, 5] and constitute one of the most frequent mental health comorbidities in the general population [6], the presence of either doubling the risk of developing the other disorder [7]. Moreover, their co-occurrence is associated with greater severity and worse prognosis than either disorder alone [8], including an increased risk for suicidal behavior [1, 9]. Published evidence has shown that about one-third of persons with a depressive disorder also meet the criteria for AUD and vice versa, especially comorbid mental problems [1, 6].

As alcohol enters the bloodstream, it affects the areas of the brain associated with emotions, resulting in exaggerated states of depression. Likewise, increased drinking to relieve depression leads to a rapid increase in the levels of alcohol in the blood and may become counter-productive. Empirical research has shown a bidirectional association between AUD and depression; both diseases can coexist, with one raising the risk of the other and aggravating it [7–9].

According to the Global Burden of Diseases, depression affected more than 300 million people worldwide in 2017. AUD accounted for 1.26% of the total Years Lived with Disability (YLD) and contributed to 8.73% of the disease burden attributed to mental and behavioral disorders [10]. Socially disadvantaged people are highly vulnerable to these disorders [11–13]. Female sex workers (FSW) are among the socially marginalized and stigmatized people likely to develop DD and AUD disorders [14, 15]. Sex work, which is defined as receiving money or goods in exchange for sexual services, is illegal in most of the world [16]. Their mental health is severely compromised by this situation, which also raises the possibility that they will use harmful substances like alcohol and other drugs to cope with this situation to get along with clients and deal with the demands of sex work daily [17, 18].

FSW are disproportionately affected by HIV and other sexually transmitted infections (STIs) [19] and unwanted pregnancies, which are often associated with unprotected (unsafe) sex and also have other negative health outcomes [20, 21]. Both DD and AUD are significantly associated with unsafe sex, including incorrect and inconsistent condom use [22], which increases the chance of getting HIV and other STIs. Generally, the occurrence of either disorder or both has serious social, economic, and short- and long-term health consequences [23].

In recent years, the burden of these disorders has been increasing in many low and low-middle-income countries (LMICs). A recent meta-analysis of AUD and depression among FSWs in LMICs estimated a pooled prevalence for harmful AUD of 40% [24] and a pooled prevalence for depression of 42% [11]. Like other low-income nations, Ethiopia had the highest prevalence of any depression (moderate to severe) and AUD, which affected 36.7% of the migrant commercial sex workers [25] and 66% of the FSWs [21], respectively. Moreover, frequently documented factors associated with depression were food insecurity, controlling partners, violence, harmful alcohol use, substance use, low income from selling sex, stigma, mobility, or lack of social support [18, 25–29], and AUD were food insecurity, violence, depression or anxiety, high income from selling sex, stigma, and police arrest [17, 21, 28, 30, 31].

FSWs constitute a multiply burdened population group, as they face high risks of DD and AUD, which contribute to HIV and other STI infections, all of which are major public health concerns. To successfully treat both of these issues, it is critical to understand the common key determinants that underpin these disorders, which is a significant knowledge gap in Ethiopia and around the world. In addressing the common key determinants, the severity level of the two disorders is very important, while previous studies felt that they employed binary outcomes, although both of the disorders are ordinal in nature. The existing researchers have so far applied a separate model to address the determinants, with one disorder being the determinant for the other disorder and vice versa. Joint modeling may facilitate and make the intervention for the two disorders more efficient.

To uncover the shared determinants for the two illnesses, taking into account their dependence, and to provide an accurate and unbiased estimate for the determinants, a joint model with ordinal outcomes will accommodate the disorders’ concealed severity. Hence, this study aimed at estimating the prevalence and common key determinants of DD and AUD among FSW in Ethiopia using bivariate multivariable ordinal logistic model. The findings of which will fill a major gap in scientific information for policy formulation and planning and targeted decision making.

## Methods

### Study design

This analysis involved the National HIV and Other STI Bio-behavioral Survey (NHSBS) data collected from six regional capital cities and 10 selected towns in Ethiopia from December 2019 to May 2020 using a respondent-driven sampling (RDS) technique. The Ethiopian Public Health Institute (EPHI) of the Ministry of Health conducted the survey with technical assistance from the United States Centers for Disease Control and Prevention (CDC) and Population Services International (PSI) in collaboration with the Federal HIV/AIDS Prevention and Control Office (FHAPCO). The NHSBS data were collected from 6085 FSWs aged 15 years and older.

### Study population and sampling procedures

Sixteen separate cross-sectional data collections were conducted using the same RDS methodology to recruit the study participants from each study site concurrently and independently. The RDS is a network-based technique for sampling hard-to-reach populations that begins with a purposive sample of seeds (the initial participant), who then recruit other members by issuing coupons provided by the investigators and are asked to invite a maximum of three additional participants from within their networks using a chain-referral procedure [32, 33]. The study sites, seeds and sample size are presented in Table 1.

**Table 1:** Sample sizes and seeds distribution by study sites, National HIV and Other STI Bio behavioral Survey (NHSBS) in Ethiopia, 2020

Study site	Sample size	Seeds	Study site	Sample size	Seeds
Addis Ababa	1101	13	Jimma	254	5
Adama	676	8	Arba Minch	251	5
Hawassa	522	8	Kombolcha/Dessie	251	5
Gambella	468	6	Dilla	251	5
Dire Dawa	434	5	Logia/Semera	251	5
Bahir Dar	372	8	Gonder	250	5
Nekemte	257	5	Shashemane	250	5
Mizan	255	5	Harar	242	5
Total				6085	98

#### Study inclusion criteria

Across all study sites, eligible participants were biologically female, aged 15 years or older, resided or worked in the survey site, sold sex for money or goods for at least four persons during the last 30 days before the survey, provided informed consent to participate in the bio-behavioral survey interview (using a questionnaire) and biological testing, and were in possession of a valid coupon.

### Study participants’ recruitment

The study started with a total of 98 seeds (initial participants) to initiate the recruitment process. The seeds were selected purposively based on the type of sex worker, age category, and geographic location of the sites. The types of sex workers considered during the selection of the seeds were bar and/or hotel-based, red-light houses, local drinking houses, street-based, and hidden (cell phone-based). Each selected seed was given three coupons to invite her friends or other FSW contacts that were in her network. The process continued similarly to recruit additional potential participants from their networks through the coupon referral system until the required sample size was achieved and the RDS equilibrium condition was attained. The coupons were active from the day that they were given to the potential participant and expired after two weeks, or when the study was completed if earlier than two weeks. An anonymous fingerprint-based code given using biometric fingerprint scanners was used to avoid multiple enrollments. The fingerprint information was not linked to the bio-behavioral questionnaire or biological test.

### Data collection and management

A standardized bio-behavioral survey questionnaire was administered in a private location to keep FSWs privacy. The data were captured directly in real-time on computer tablets using open data kit (ODK) electronic data software with built-in skip patterns and logical validations and monitored daily to ensure data quality. The RDS assumptions were monitored during data collection using the RDS package built into R statistical software [34]. The survey collected information on socio-demographic, HIV and other STI prevalence, DD and AUD, sexual behaviors, reproductive health, and participants’ engagement in HIV prevention and treatment services.

### Study variables

Outcome variables: The analysis involved two outcome (dependent) variables. The first was the DD severity level computed from the Patient Health Questionnaire (PHQ9) assessment tool [35]. Participants were asked to score nine common symptoms of DD in the past two weeks, with responses of “not at all” (= 0), “several days” (= 1), “more than half the days” (= 2), and “nearly every day” (= 3), with item scores summed to produce a total score range of 0–27. A score of 0–4 was labeled as “non-minimal”, 5–9 as “mild”, 10–14 as “moderate”, 15–19 as “moderately severe,” and 20–27 as “severe” DD severity levels (alpha = 0.84). A PHQ-9 score ≥ 10 was considered as major DD (alpha = 0.88).

The second outcome variable was AUD, which represents alcohol dependence and alcohol addiction. The alcohol dependence level was computed from the Alcohol Use Disorder Identification Test (AUDIT) [35]. The AUDIT uses 10 items to provide an assessment of AUD. Participants responded to items on response scales, ranging from 0 (never) to 4 (four or more times a week) for items 1–8, and from 0 (no) to 2 (yes, during the last year) for items 9 and 10, with item scores summed to produce a total score range of 0–40. A score of 0–8 labeled as “social drinking”, 9–13 “harmful or hazardous drinking”, and 13–40 for “alcohol dependence” severity levels has been recommended for alcohol dependence (alpha = 0.75).

Independent Variables: The independent variables included in the analysis to identify the common determinants of major DD and AUD were socio-demographic (age, marital status, education, sex work location, and income), sex work experience (year sold sex, age started sex, and age started selling sex), violence, two STI symptoms, abortion, behavioral characteristics (smoking cigarettes, chewing Khat and any drug use (tobacco and injectables (heroin, opiates, and cocaine))), number of sexual partners(both paying and non-paying partners in the last 6 months), history of pregnancy (abortion and miscarriage), history of unsafe sex (not used and slip/break of condom) and HIV test result.

### Statistical analysis

Statistical analysis was conducted using STATA v.16 and R v.3.6.2. The RDS recruitment process (Tree of recruitment), assessment of the RDS assumptions, and RDS weight generation were implemented using the RDS package inbuilt in R statistical software [34]. Homophily and convergence were the common assumptions in RDS and were checked in HIV status, consistent condom use, and type of FSW and met the RDS criteria. The RDS weights were exported using the RDS-II function to STATA and merged with the whole dataset for further analysis. Descriptive statistics like the crude and RDS-adjusted frequency, mean, and standard deviation were calculated using STATA.

The bivariate multivariable ordinal logistic regression (BMOLR) model was used to jointly estimate the non-normal correlation between DD and AUD, as well as the determinants of each. We used multivariate joint modeling because it has an advantage over separate modeling in its relative efficiency to estimate the model [36, 37]. It also has an ability to accommodate the contributions of the determinants to the correlation between the outcome variables and provides a powerful test of significance compared to univariate techniques [38].

The BMOLR model used a composite likelihood estimation technique to estimate the dependence between ordinal measurements on the same subject and the associated factors for each measurement simultaneously [39]. Since our data on DD consisted of four measurements (none, mild, moderate, and moderately severe), and alcohol consumption level consisted of three categories (social drinking, harmful or hazardous, and dependence), the BMOLR model provided the non-normal correlation of DD and alcohol consumption and the odds ratio with 95% confidence interval (CI) of the determinants of DD and alcohol consumption. The statistical significance of estimates for single variables and associations between determinants and outcomes was determined based on a 95% CI that didn’t include unit or overlap as appropriate.

### Ethical consideration

The Ethiopian Public Health Institute’s (EPHI) Scientific and Ethical Research Office (SERO) granted ethical approval for the study protocol (Ref. EPHI 6¢13/517). Using the local language (Amharic and Afan Oromo), potential participants were told about the study’s objective and methods, potential hazards, and safeguards. Each survey participant provided written informed consent for the interview, blood sample collection, and storage of biospecimens for future testing.

## Results

### Prevalence of major DD and AUD

**Fig. 1:**
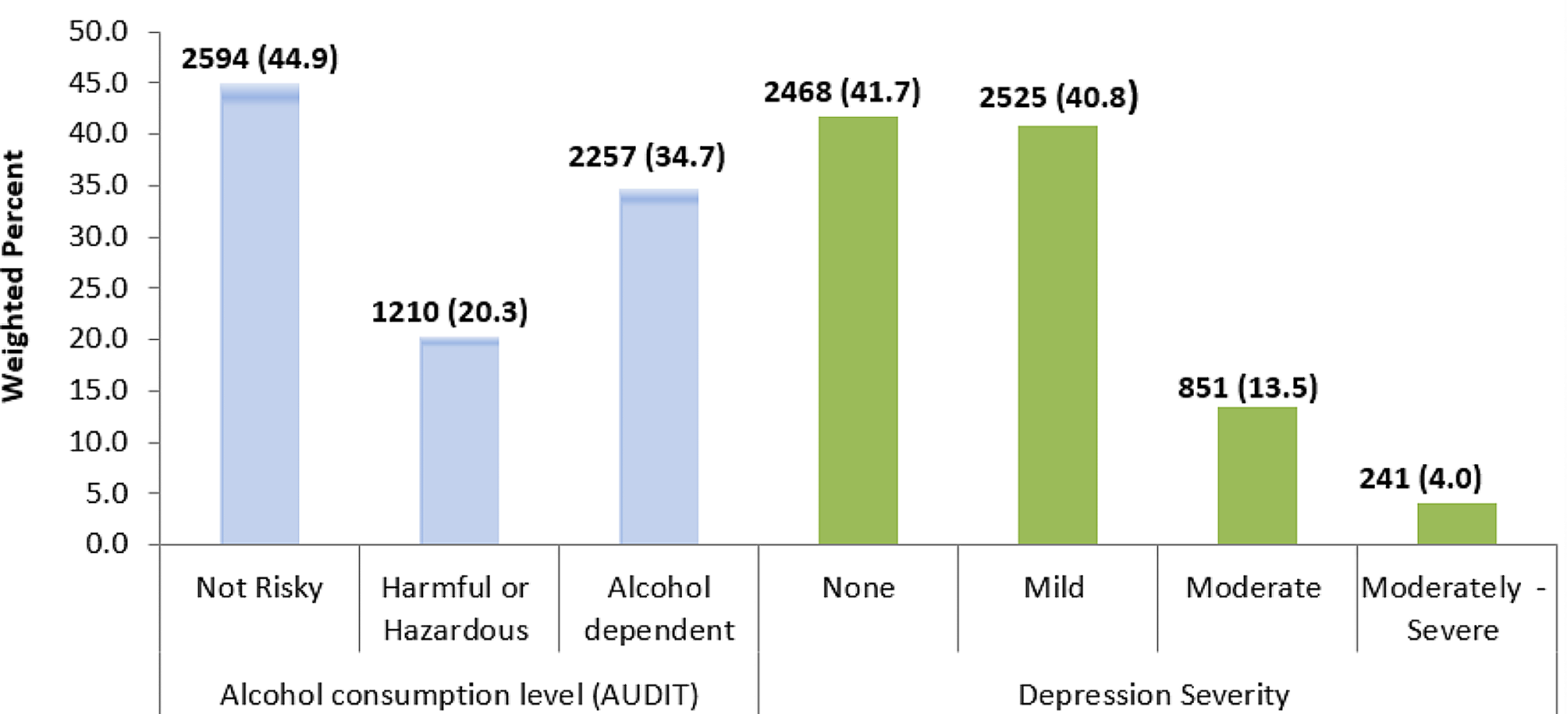
Weighted prevalence of alcohol consumption level (AUDIT) and DD severity (PHQ-9) among female sex workers, Ethiopia, 2020 (*N* = 6085)

Among the 6085 participants, 1210 (20.3%) and 2257 (34.7%) met the AUDIT criteria for harmful or hazardous and an indication of alcohol dependence, respectively, resulting in total in 3467 (55.0%) of hazardous or dependent alcohol consumption level. Of the total participants, 851 (13.5%) and 241 (4.0%) met the DD screening of PHQ-9 criteria of moderate and moderately-severe DD, respectively, resulting in total in 1092 (17.5%) major DD level (Fig. 1).

### Prevalence and characteristics of major DD and hazardous or more AUD

The mean age (SD) of the study participants was 26.3 (+ 6.1) years. The prevalence of major DD and hazardous or more AUD significantly varied across the sixteen study sites; the lowest prevalence of major DD was 5.4 (95% CI: 3.2–8.6) in Nekemte and the highest was 41.6 (95% CI: 37.4–46.1) in Gonder, and AUD was 34.9 (95% CI: 31.4–38.5) in Logiya/Semera and the highest was 80.4 (95% CI: 77.4–83.2) in Dilla. The prevalence of major DD significantly increased with increasing age categories, with the lowest being 15.8% in the age category 15–24 years and the highest being 21.3% in the age category 35–59 years. Conversely, the lowest prevalence (46.3%) of AUD was in the older age category, 35–59 years (Table 2).

As is also shown in Table 2, the proportion (21.0%) of those screened for major DD was significantly higher among FSW with no education, compared with 16.7% among those with primary or more education. The proportion of those who met the criteria for hazardous or more drinking significantly increased as the educational level increased; the lowest was 41.0% in those with no formal education, and the highest was 60.3% in those with secondary school education or more. FSW who received an average monthly income of less than $200 from selling sex had a higher prevalence 19.3% (95% CI: 18.5–20.1) of major DD and a lower AUD 50.2% (95% CI: 49.2–51.3) than those who received more than USD 200, with a proportion of 13.8% (95% CI: 12.8–14.8) and 64.9% (95% CI: 63.5–66.3), respectively.

### Prevalence of major DD and AUD by behavioral and other characteristics

As summarized in Table 3, FSW forced to have sex for the first time had significantly higher prevalence than those who were not forced of major DD and hazardous or more AUD, 23.1% (95% CI: 21.5–24.6) vs. 15.9 (95% CI: 15.2–16.6) and 58.7% (95% CI: 56.9–60.6) vs. 54.0% (95% CI: 53.0–55.0), respectively. Likewise, FSWs who reported the presence of STI symptoms and were raped or forced to have sex in the last 12 months before the start of data collection had significantly higher prevalence of major DD, 26.3% (95% CI: 24.5–28.2) and 30.4% (95% CI: 28.3–32.6), and hazardous or more AUD, 67.4% (95% CI: 65.3–69.3) and 77.0% (95% CI: 74.9–78.9), respectively, compared with their counterparts who did not have STI symptoms and were not raped or forced to have sex in the last 12 months, 15.8% (95% CI: 15.1–16.5) and 52.7% (95% CI: 51.7–53.6).

**Table 2:** Weighted prevalence of major DD and AUD by background characteristics among female sex workers, Ethiopia, 2020 (*N* = 6085)

Variables	Major DD		AUD		Total
	Percent	95% CI	Percent	95% CI	
Study site					
Adama	12.7	11.1–14.5	79.3	77.3–81.3	676
Addis Ababa	9.9	8.9–11.1	40.1	38.3–42	1101
Arba Minch	15.5	12.9–18.4	44.7	40.9–48.4	251
Bahir Dar	23.7	20-27.9	35.1	30.7–39.6	372
Combolcha/Dessie	5.8	3.4-9	51.3	45.1–57.2	251
Dilla	37.4	33.9–40.9	80.4	77.4–83.2	251
Diredawa	10.4	8.5–12.4	67.1	64–70	434
Gamebella	28.4	24.4–32.5	61.1	56.6–65.3	468
Gonder	41.6	37.4–46.1	52.7	48.2–57	250
Harar	7.9	6.2–10	46.4	42.8–49.9	242
Hawassa	19	16.3–22	60	56.4–63.5	522
Jimma	39.6	36.2–42.9	69.2	65.9–72.4	254
Logia/Semera	3.9	2.7–5.6	34.9	31.4–38.5	251
Mizan	21.6	18.8–24.7	37.5	34.1–41	255
Nekemite	5.4	3.2–8.6	64	58.2–69.5	257
Shashemane	20.6	17.7–23.7	62.7	59.2–66.2	250
Age category					
15–24	15.8	14.9–16.8	56.4	55.1–57.7	2595
25–34	17.9	16.9–18.9	56.6	55.3–57.9	2671
35–59	21.3	19.5–23.2	46.3	44-48.6	819
Marital status					
Married/Cohabitation	16.4	13.5–19.9	61.3	57-65.4	231
Divorced/Separated/Widowed	18.5	17.5–19.5	51.4	50.2–52.7	2908
Never married	16.5	15.6–17.5	58.1	56.9–59.3	2946
Highest grade attended					
Non-formal Education	21	19.4–22.7	41	39–43	1054
Primary school (grade 1–8)	16.7	15.8–17.5	57.5	56.3–58.6	3560
Secondary school and above	16.6	15.3–17.9	60.3	58.6–62	1471
Main source of income					
other than sex work	15.8	13.6–18.2	42.6	39.5–45.7	391
Sex work	17.6	16.9–18.3	56.1	55.2–56.9	5694
Average monthly income from selling sex in USD					
Less than $200	19.3	18.5–20.1	50.2	49.2–51.3	3844
$200 and more	13.8	12.8–14.8	64.9	63.5–66.3	2241
Years lived in the current city/place					
< 5 Years	16.5	15.5–17.5	53.2	51.8–54.6	2308
5–10 Years	15.3	14.1–16.6	56.1	54.4–57.8	1559
11 + Years	20	18.9–21.2	56.3	54.9–57.8	2218

As Table 3 revealed in terms of 30 days behavioral risk variables, the proportion of FSW who were smoking cigarettes or cigars and met the screening criteria for major DD and hazardous or more AUD of 21.5% (95% CI: 19.4–23.7) and 82.7% (95% CI: 80.6–84.6) was significantly higher than those who were not smoking, 17.0% (95% CI: 16.3–17.70) and 51.7% (95% CI: 50.8–52.7). The prevalence of major DD and hazardous or more AUD among FSW who used any other drugs was 22.8% (95% CI: 20.7–25.1) and 83.1% (95% CI: 81.0–85.0), which was significantly higher than in those who did not use any other drugs, 16.8% (95% CI: 16.1–17.5) and 51.7% (95% CI: 50.8–52.6), respectively.

Among FSW with a history of miscarriage and abortion, the prevalence of major DD was 24.4% (95% CI: 22.2–26.7) and 20.8% (95% CI: 19.3–22.4) and hazardous or more AUD was 61.7% (95% CI: 59.2–64.3) and 71.3% (95% CI: 69.6–73.1), respectively, which was significantly higher than those not having a history of miscarriage and abortion. The prevalence of major DD and hazardous or more AUD was significantly higher in FSW who inconsistently used condoms during the last 30 days: 23.2% (95% CI: 21.5–25.0) and 71.1% (95% CI: 69.2–73.0), respectively; in those who experienced condom failure or slipper, 24.0% (95% CI: 22.7–25.4) and 66.5% (95% CI: 65.1–68.1); and in those who ever had anal sex, 23.5% (95% CI: 20.9–26.1) and 74.7% (95% CI: 71.9–77.3). The prevalence of AUD significantly increased with the number of sites the FSW worked selling sex in the last 3 years: 50.2% (95% CI: 49.3–51.2%), 70.3% (95% CI: 68.0-72.5), and 87.2% (95% CI: 84.7–89.3) for the same, one more, and two or more cities or towns, respectively. FSW who had fewer than 31 paying clients during the last six months had the highest prevalence of major DD, at 20.7% (95% CI: 19.7–21.8), and the lowest prevalence, at 48.4% (95% CI: 47.–49.7), of hazardous or increased AUD. The prevalence of DD and AUD among FSW significantly increased with the number of non-paying partners in the last 6 months, from 16.0 (95% CI: 15.2–16.7) to 26.5% (95% CI: 23.7–29.6) for DD and from 50.0% (95% CI: 49.0–51.0) to 75.8% (95% CI: 72.9–78.6) for AUD, respectively, among those who had none and more than two nonpaying partners (Table 3).

### Correlation of DD Severity and ADU

The BMOLR model revealed that the non-normal correlation between general DD severity and AUD was moderately positive (*r* = .22; 95% CI: 0.19, 0.25), indicating an increase in one problem increases the other one (Table 4).

### Determinants of DD Severity and ADU

After adjusting for potential confounding factors, FSW who had HIV positive status and anal sex experience had (AOR = 1.26; 95% CI: 1.11–1.44) and (AOR = 1.31; 95% CI: 1.08–1.12), i.e., 1.26 and 1.31 times the odds of reporting an increased DD severity compared with their HIV positive and no anal sex experience counterparts, respectively (Table 4). FSWs who attended primary school (AOR = 1.69; 95% CI: 1.46–1.95) and secondary education (AOR = 1.76; 95% CI: 1.49–2.08) were 1.69 and 1.76 times more likely to increase the alcohol dependence level, respectively, as compared to those who did not attend formal education. FSW with average monthly income from selling sex who earned $200 or more had DD severity reduced by 18% (AOR = 0.82; 95% CI: 0.74–0.92) and alcohol dependency increased by 33% (AOR = 1.33; 95% CI: 1.19–1.49) compared with those earning less than $200. Among FSW who worked for over five years, the odds of reporting an increase in DD severity was 1.13 (95% CI: 1.15–1.49) times and the odds of reporting an increase in alcohol dependence was 1.33 (95% CI: 1.33–1.75) times when compared with those who worked for less than three years, respectively. Those who worked sex selling in two or more cities or towns during the last three years preceding the survey had 1.47 (95% CI: 1.19–1.81) and 3.11 (95% CI: 2.45–3.95) times the odds of reporting an increase in DD severity and alcohol dependence, respectively, compared with those working in the same town.

**Table 3:** Weighted prevalence of major DD and AUD by sexual risk behaviors and non-consensual sex among female sex workers, Ethiopia, 2020 (*N* = 6085)

Variables	Major DD		AUD		Total (*n*)
	Percent	95% CI	Percent	95% CI	
Age at first sex selling					
Less than 20	18.4	17.3–19.5	60	58.7–61.4	2328
20–24	16.1	15.1–17.2	56.2	54.8–57.6	2348
25+	18.1	16.8–19.5	45.4	43.7–47.1	1406
Age at first sex					
15 or less	18.5	17.5–19.6	56.4	55-57.8	2430
16–20	16.5	15.6–17.3	55	53.9–56.2	3384
21+	20.2	17.4–23.3	45.8	42.1–49.5	271
First sex experience					
Wanted	15.9	15.2–16.6	54	53–55	4738
Forced	23.1	21.5–24.6	58.7	56.9–60.6	1347
At least Two STI symptoms occurred in the last 12 months					
No	15.8	15.1–16.5	52.7	51.7–53.6	5083
Yes	26.3	24.5–28.2	67.4	65.3–69.3	1002
Ever been raped or forced to have sex in the past 12 months					
No	15.5	14.8–16.1	51.7	50.8–52.6	5314
Yes	30.4	28.3–32.6	77	74.9–78.9	771
History of miscarriage pregnancy					
No	16.6	16-17.3	54.3	53.4–55.2	5471
Yes	24.4	22.2–26.7	61.7	59.2–64.3	614
History of aborted pregnancy					
No	16.6	15.9–17.3	51	50-51.9	4809
Yes	20.8	19.3–22.4	71.3	69.6–73.1	1276
In the last 30 days, used cigarettes or cigars					
No	17	16.3–17.7	51.7	50.8–52.7	5343
Yes	21.5	19.4–23.7	82.7	80.6–84.6	742
Chewing khat in the last 30 days					
Never	17.4	16.4–18.5	35.8	34.5–37.2	2258
Yes	17.5	16.7–18.3	66.7	65.7–67.7	3827
Used any drugs in the last 30 days					
Never	16.8	16.1–17.5	51.7	50.8–52.6	5385
Yes	22.8	20.7–25.1	83.1	81–85	700
Number of cities worked sex selling in the last three years					
Same town	16.8	16.1–17.5	50.2	49.3–51.2	4933
1 more town	18.9	17-20.9	70.3	68-72.5	776
2 or more towns	23.5	20.7–26.4	87.2	84.7–89.3	374
Consistently condom used in the last 30 days					
No	23.2	21.5–25	71.1	69.2–73	966
Yes	16.3	15.6–17	51.8	50.8–52.7	5119
Experienced condom failure/slipped in the last 30 days					
No	14.8	14-15.5	50.3	49.3–51.3	4260
Yes	24	22.7–25.4	66.6	65.1–68.1	1825
Ever had anal sex					
Never	17	16.3–17.6	53.4	52.5–54.3	5659
Yes	23.5	20.9–26.1	74.7	71.9–77.3	426
Number of paying partners in the past 6 months					
4–30	20.7	19.7–21.8	48.4	47.1–49.7	2308
31–90	16.9	15.8–18.1	58.4	56.9–59.9	2132
91+	12.2	11.1–13.4	62.8	61.1–64.5	1645
Number of non-paying partners in the past 6 months					
Never	16	15.2–16.7	50	49–51	4347
Only one	19.7	18.3–21.2	65.9	64.1–67.6	1404
2 and more	26.5	23.7–29.6	75.8	72.9–78.6	334

As is also summarized in Table 4, FSW who used any other drugs (AOR = 1.35; 95% CI: 1.16–1.58) were more likely to report an increase in DD severity and were over three times more likely (AOR = 3.32; 95% CI: 2.79–3.95) to report an increase in hazardous or more AUD, respectively, compared with those who did not use other drugs. FSW who used condoms inconsistently, experienced condom failure, were raped or had forced sex, and self-reported two STI symptoms were more likely to report an increase in DD severity (AOR = 1.24; 95% CI: 1.1–1.4), (AOR = 1.5; 95% CI: 1.34–1.68), (AOR = 2.22; 95% CI: 1.91–2.31), and (AOR = 1.71; 95% CI: 1.5–1.95), respectively, and hazardous or more AUD (AOR = 1.97; 95% CI: 1.74–2.23), (AOR = 1.44; 95% CI: 1.28–1.62), (AOR = 2.31; 95% CI: 1.96–2.73), and (AOR = 1.36; 95% CI: 1.18–1.56), respectively, compared to their counterparts who did not report these characteristics. The odds of reporting increased DD severity and hazardous or more AUD in those who had experienced abortion were 1.26 (95% CI: 1.11–1.43) and 1.53 (95% CI: 1.34–1.74) times higher than those who had no abortion, respectively.

As is also shown in Table 4, FSWs who had over 90 paying clients in the past 6 months (AOR = 0.86; 95% CI: 0.82–0.89) were less likely by 14% to report an increase in severe DD compared to those who had 30 or fewer clients. As indicated by the AOR, FSW who had over 90 paying clients in the past 6 months were 1.14 (95% CI: 1.1.09–1.19) times more likely to have hazardous or more AUD, and those who had only one or two and more nonpaying partners were 1.26 (95% CI: 1.12–1.42) and 1.53 (95% CI: 1.23–1.9) times more likely to have an increased DD severity, and 1.66 (95% CI: 1.47–1.88) and 2.07 (95% CI: 1.63–2.61) times more likely to have increased hazardous or more AUD in the past six months compared with their reference counterparts, respectively. Those who started selling sex before the age of 20 years and at 20–24 years were 1.5 (95% CI: 1.31–1.72) and 1.27 (95% CI: 1.11–1.45) times the odds of reporting higher hazardous or more AUD compared with those who started at 25 or more years.

**Table 4:** Bivariate final ordinal regression model outputs for the determinants of the dependence between DD severity and alcohol dependence severity among female sex workers, Ethiopia, 2020 (*N* = 6056)

Variables	DD		AUD	
	OR	95% CI	OR	95% CI
Intercept				
Cutoff 0|1	0.72**	0.6–0.86	5.08**	4.15–6.21
Cutoff 1|2	5.48**	4.55–6.6	13.46**	10.93–16.57
Cutoff 2|3	30.75**	24.67–38.32		
HIV Test Result				
Negative	1		1	
Positive	1.26**	1.11–1.44	0.96	0.84–1.1
Highest grade attended				
Non-formal Education	1		1	
Primary school (grade 1–8)	0.93	0.82–1.06	1.69**	1.46–1.95
Secondary school and above	0.97	0.83–1.13	1.76**	1.49–2.08
Average monthly income from selling sex in USD				
Less than $200	1		1	
$200 and more	0.82**	0.74–0.92	1.33**	1.19–1.49
Number of years lived as FSW				
< 3 Years	1		1	
3–5 Years	1.03	0.92–1.16	1.32**	1.18–1.49
6 + Years	1.31**	1.15–1.49	1.53**	1.33–1.75
Used any drugs				
Never	1		1	
Yes	1.35**	1.16–1.58	3.32**	2.79–3.95
Anal sex experience				
No	1		1	
Yes	1.31**	1.08–1.6	1.12	0.91–1.37
Number of cities worked sex selling in the last three years				
Same town	1		1	
1 more town	1.12	0.96–1.3	1.86**	1.6–2.18
2 or more towns	1.47**	1.19–1.81	3.11**	2.45–3.95
Number of paying partners in the past 6 months				
4–30	1		1	
31–90	1.04	0.94–1.15	1.06	0.95–1.18
91+	0.86**	0.82–0.89	1.14**	1.09–1.19
Number of non-paying partners in the past 6 months				
Never	1		1	
Only one	1.26**	1.12–1.42	1.66**	1.47–1.88
2 and more	1.53**	1.23–1.9	2.07**	1.63–2.61
Two STI symptoms occurred in the last 12 months				
No	1		1	
Yes	1.71**	1.5–1.95	1.36**	1.18–1.56
Ever been raped or forced to have sex in the past 12 months				
No	1		1	
Yes	2.22**	1.91–2.59	2.31**	1.96–2.73
Consistently condom used in the last 30 days				
No	1.24**	1.1–1.4	1.97**	1.74–2.23
Yes	1		1	
Experienced condom failure in the last 30 days				
No	1		1	
Yes	1.5**	1.34–1.68	1.44**	1.28–1.62
Age at first sex selling				
< 20	1.08	0.95–1.23	1.5**	1.31–1.72
20–24	0.94	0.83–1.07	1.27**	1.11–1.45
25+	1		1	
History of aborted pregnancy				
No	1		1	
Yes	1.26**	1.11–1.43	1.53**	1.34–1.74
Correlation	*r* = .22 95% CI: (0.19, 0.25)			

Note: * *p* < .05; ** *p* < .01

## Discussion

This is the first study on FSW that used RDS and included as many as 16 major cities/towns in Ethiopia, which assessed the prevalence and determinants of major DD and AUD combined. The results show that one in every six (17.5%) of FSW in Ethiopia had major DD in 2020, of which 13.5% were moderate and 4.0% severe. In our study, the prevalence of major DD among FSW was higher than the prevalence among the overall adult population of Ethiopia (moderate DD 4.2% and severe DD 2.5%) [40], the prevalence among urban migrants (4.7%), and the prevalence among urban migrants working as FSW (9.8%) [25]. The greater prevalence of DD in the current study could potentially be attributed to the fact that FSWs are more likely than the general population to experience significant DD. This prevalence identified in our study is similar to that reported among the FSW in Cameroon [41] and Malawi [42], but lower than what has been reported from Kenya [43], Switzerland [44] and India [28]. The lower prevalence of DD in the current study could be attributed to the sampling process and sample size used. The current study used RDS, while the others used traditional cross-sectional, and the current study had about three times the sample size of the others.

Likewise, one in every two FSW (55.0%) in our study had AUD, of which 20.3% and 34.7% were harmful/hazardous and had an alcohol dependence indicating level of alcohol consumption, respectively. The prevalence of AUD is higher compared with that in other population groups in Ethiopia: urban adult population (13.1%), patients with epilepsy (17.1%), and patients with schizophrenia (38.4%) [45–47], but it concurs with the finding from another study in Ethiopia [48] and the report among FSW in Malawi [49]. The higher prevalence of AUD in the current study could potentially be attributed to the fact that FSWs are more likely than the general population to experience significant AUD.

The BOLR model was employed to jointly estimate the non-normal correlation between DD and AUD severity and their determinants among FSW. The results from this combined estimation would contribute to reducing debates on the direction of the causal relationship between major DD and AUD. Accordingly, the findings from the model indicate that an increase in DD severity increased the severity of AUD and vice versa, indicating a moderately positive correlation between major DD and AUD. Our finding was consistent with that found in street FSWs in Addis Ababa, Ethiopia [48], and among the general population elsewhere [4, 8, 50].

### Factors commonly associated with DD and AUD

An average monthly income of USD 200 or more made by FSW from selling sex decreased DD and increased AUD severity compared with less than USD 200. This supports the findings of a study conducted in the Dominican Republic, which showed that low income from selling sex was associated with worse DD [51], and reports from Ethiopia and Malawi indicating that AUD facilitates FSW’s solicitation with clients and helps in price negotiation [21, 49, 52]. Overall, these results suggested that having enough money could help fight DD and reduced alcohol consumption may serve as the first step towards greater economic security [31].

In our study, FSWs engaging in sex work for more than five years were more likely to report severe DD and AUD than those engaging for less than three years. The fact that FSWs engage in sex-selling work could result in stigmatization, discrimination, and social isolation by the community, behaviors that are associated with DD [21, 49]. DD and AUD seem to have a bidirectional relationship [8]. DD severity increasing the risk for AUD, and more engagement in selling sex might increase the severity of DD, which in turn increases AUD severity.

This study also adds to previous findings that show FSW who experienced violence (sexual and/or physical) during the 12 months preceding the survey were more likely to report severe DD and AUD than others. Reports from previous studies show consistent results that indicate FSW experiencing violence are at a higher risk of DD [25–29] and AUD [31]. The findings of our study suggest that programmatic attention through awareness creation about the factors that increase the likelihood of violence is needed to protect FSW.

The findings of our study confirm that mobility of FSW from one work site to another is significantly associated with reported severe DD and AUD, the severity rates of which are higher among FSW who sold sex in more than three cities/towns during the three years preceding the study. Concurring with reports from previous studies, mobility may not be directly associated with the severity of DD or AUD but may rather escalate FSW experiencing violence as a result of selling sex in new environments and the lack of social support [28, 53]. Mental health correlates with AUD [26, 54] and leads to alcohol dependence. These multiple vulnerabilities of FSW are a serious concern and require urgent interventions.

Our findings also show that FSW who have had more than 90 paying partners during the last six months have a decreasing rate of DD and an increasing severity of alcohol dependence. An Indian study showed that DD has no association with the number of clients in the previous one month [55]. Likewise, FSWs with more than one non-paying partner were more likely to report an increase in DD and alcohol dependence severity than those who had none. Another Indian study found that FSW who have unpaid partners are more violent [28] and that violence causes mental health issues, which is also supported by our findings.

FSW who reported condom breakage/slipping and inconsistent condom use have an increasing rate of severe DD and alcohol dependence, which constitutes a major concern for HIV/sexually transmitted infection prevention programs. The findings of our study also highlighted that FSW’s history of STIs during the last 12 months was associated with increased severity of DD and alcohol dependence. This concurs with a report from South Africa [56] and AUD [57]. Similarly, FSW with a history of abortion had more severe DD and alcohol dependence, a finding that is consistent with a report from China that showed that abortion was associated with a high rate of DD [58]. This may be explained by inconsistent condom use, condom use failure, the presence of violence, or a high number of nonpaying partners. Our findings also suggested that severely depressed people and alcohol abuse had outcomes of inconsistent condom use and failure of condom during the last 30 days.

### Factors specifically Associated with DD and AUD

Our finding suggested that HIV-positive FSW had an increased risk of severe DD but not AUD compared with HIV-negative FSW. Previous studies have also shown an association between HIV-positive status and severe DD ( [18, 26], contrasting the finding of a study report from South Africa [56]. HIV infection, like the finding from another study in Ethiopia [21], was not significantly associated with alcohol dependence in our study, which differs from studies elsewhere that reported a significant association of HIV with high alcohol consumption [49, 59]. Our study also showed that FSW who started selling sex before the age of 25 years have increased consumption of alcohol, a finding that is consistent with a previous study in Ethiopia [21]. Our observation related to anal sex requires further investigation through further research, as this is a gap in the available literature.

### Strength and limitation of the study

Unlike most previous studies that used nonprobability sampling methods among FSW, we selected a representative sample of FSW using a respondent-driven sampling technique across 16 regional capitals and major towns in Ethiopia. Thus, the study’s findings are more generalizable to similar contexts. Also, unlike most previous studies that only screened for depressive symptoms or AUD independently, we screened using PHQ-9 criteria for DD and AUDIT for alcohol. We believe that this approach fosters a better understanding of the burden of the problem associated with these outcomes combined. Moreover, the joint multivariate model identified the non-normal correlation among major DD and AUD and key determinants. This makes our approach unique in providing a better understanding of how the two disorders combine. The information collected have been influenced by recall bias since we asked FSW about their past histories. Nonetheless, most of the information asked was for events within two weeks before the interviews for DD and 30 days for the AUDIT, thus reducing the possibility of a recall bias.

## Conclusion

This present study highlights that major DD and AUD are prevalent among FSW in Ethiopia. The average monthly income from selling sex, use of any drugs, mobility, sold sex for more than five years, number of paying and nonpaying partners, inconsistent condom use, condom failure, history of STI symptoms and abortion, and violence were statistically significant determinants of major DD and alcohol dependence. Besides making condoms available at work places and provision of education on the consistent and correct use of condoms to reduce HIV, other STIs, and unwanted pregnancies, integrated STI strategies need mental health and AUD screening, referral and linkage to services, and treatment for FSW. Governments and agencies working with FSW need to highlight the barriers to addressing mental health and AUD behaviors, initiate counseling services, and provide information on mental health and alcohol consumption strategies and associated risk factors. Sex work is unlawful and socially unacceptable in society, exacerbating the issue of DD and AUD. The community and those working in psychosocial support could counsel FSWs to refrain from engaging in this type of activity by raising awareness and providing economic empowerment.

## Data Availability

The datasets used and/or analysed during the current study available from the corresponding author on reasonable request.

## Declarations

In accordance with EPHI institutional review board approval, all study participants provided informed consent to give a blood sample and voluntarily participate in the study. Participants consented as per the EPHI IRB protocol, confirmed their willingness to participate by signing, and were monitored by trained supervisors during the data collection process. Before conducting study-specific procedures, we obtained consent from each participant using local languages. Volunteers were given the option to consent in their preferred language.

Participants received a signed informed consent form for their records. All participants agreed to sample storage prior to participation. The consent forms clearly explained that participants had the right to withdraw consent at any point during the study. Additionally, the consent forms detailed the study procedures and the associated risks and benefits of participating.
